# CellGAT: A GAT-Based Method for Constructing a Cell Communication Network Integrating Multiomics Information

**DOI:** 10.3390/biom15030342

**Published:** 2025-02-27

**Authors:** Tianjiao Zhang, Zhenao Wu, Liangyu Li, Jixiang Ren, Ziheng Zhang, Jingyu Zhang, Guohua Wang

**Affiliations:** 1 College of Computer and Control Engineering, Northeast Forestry University, Harbin 150040, China; tianjiaozhang@nefu.edu.cn (T.Z.); 2022112562@nefu.edu.cn (Z.W.); liangyuli@nefu.edu.cn (L.L.); 1241126773@nefu.edu.cn (J.R.); zzhjs@nefu.edu.cn (Z.Z.); 2 Department of Radiology, The Second Affiliated Hospital of Harbin Medical University, Harbin 150040, China; hmuzjy@hrbmu.edu.cn; 3 Faculty of Computing, Harbin Institute of Technology, Harbin 150001, China

**Keywords:** cell–cell interactions, scRNA-seq, deep learning, graph convolutional neural network, graph attention networks

## Abstract

The growth, development, and differentiation of multicellular organisms are primarily driven by intercellular communication, which coordinates the activities of diverse cell types. This cell-to-cell signaling is typically mediated by various types of protein–protein interactions, including ligand–receptor; receptor–receptor, and extracellular matrix–receptor interactions. Currently, computational methods for inferring ligand–receptor communication primarily depend on gene expression data of ligand–receptor pairs and spatial information of cells. Some approaches integrate protein complexes; transcription factors; or pathway information to construct cell communication networks. However, few methods consider the critical role of protein–protein interactions (PPIs) in intercellular communication networks, especially when predicting communication between different cell types in the absence of cell type information. These methods often rely on ligand–receptor pairs that lack PPI evidence, potentially compromising the accuracy of their predictions. To address this issue, we propose CellGAT, a framework that infers intercellular communication by integrating gene expression data of ligand–receptor pairs, PPI information, protein complex data, and experimentally validated pathway information. CellGAT not only builds a priori models but also uses node embedding algorithms and graph attention networks to build cell communication networks based on scRNA-seq (single-cell RNA sequencing) datasets and includes a built-in cell clustering algorithm. Through comparisons with various methods, CellGAT accurately predicts cell–cell communication (CCC) and analyzes its impact on downstream pathways; neighboring cells; and drug interventions.

## 1. Introduction

Intercellular interactions are crucial in many biological processes, including cell growth, division, differentiation, tissue or organ development, and even play significant roles in disease progression. The study of single ligand–receptor pairs and actual cell–cell communication represents a progressive exploration from the molecular to the systemic level. Research on single pairs provides foundational data for understanding cell–cell communication, while the analysis of cell–cell communication further enhances our comprehension of the overall functionality of biological systems. The signaling of CCC is typically mediated by various types of PPIs, including ligand–receptor (LR), receptor–receptor, and extracellular matrix–receptor interactions. Understanding multicellular organisms and their functions requires a deep understanding of cellular activities, often achieved through studying signaling events-induced responses and downstream effects [1]. These signaling events include the binding of secreted ligands to homologous receptors, as well as intercellular [2] or intracellular ligand–receptor interactions. Understanding the phenotypic effects of these cell–cell communication patterns under healthy, perturbed, and diseased conditions is increasingly recognized [3]. Changes in intercellular communication are crucial for understanding tissue function and disease progression [4], and understanding changes in intercellular communication is crucial for deciphering tissue function and disease progression, particularly in tumor advancement, where direct cell–cell communication plays a critical role [5]. Therefore, using computational tools to simulate this cell–cell communication role in tumors can help identify biomarkers for disease severity in patients and improve drug responses in treatment approaches [6].

PPIs on the cell surface facilitate the efficient exchange of information between cells or between cells and their environment, occurring in a highly dynamic, environment, time, and space-dependent manner [7]. However, identifying interactions in native microenvironments often requires expensive experiments or extensive domain knowledge to detect active interactions [8]. To seek relatively facile methods for studying CCC, researchers have developed numerous computational approaches such as CellPhoneDB [9], Crosstalkr [10], NicheNet [11], and CellChat [12]. These methods are all gene ligand–receptor expression information and downstream target analysis, paving new paths for exploring cell–cell communication at the single-cell level. Building upon these methods, researchers have extensively applied approaches to studying CCC in different models, for instance, spatial transcriptomic data, where cellular coordinate data can better visualize CCC predictions and provide the basis for spatial adjacency. However, few methods are considering the importance of PPIs in intercellular communication networks. The absence of PPI information may lead these methods to inaccuracies, as abnormal gene expression of certain genes might not directly translate to loss of activity in the encoded proteins, affecting the accuracy of predictions.

To achieve more accurate predictions of CCC and enhance the biological relevance of its results, there are still several limitations that need to be addressed. For instance, there lacks a unified ground truth for validated LR interactions, and many datasets lack annotations such as protein complex information and pathway information involved in LR interactions. Previous methods have shown that integrating both ligand and receptor expression information with context-specific transcriptomic data affects the accuracy of predicting LR interactions based solely on expression values. Therefore, this study integrates gene expression information of ligands and receptors, PPI information, protein complex information, and experimentally validated pathway information to improve the accuracy of existing methods in predicting CCC. To address the challenge of annotating multiple large dataset inputs and projecting them onto smaller test sets, this study proposes the use of graph attention neural networks as potential solutions for predicting CCC. The introduction of this new CCC prediction framework will aid in the discovery of new ligand–receptor interactions, which may reveal novel cellular functions or roles under different environments.

To address these challenges, this paper proposes CellGAT, a deep learning approach based on graph attention neural networks for predicting cell–cell communication in scRNA-seq data. CellGAT integrates ligand–receptor annotations (such as protein complexes, PPIs, cell type, and pathway information) along with gene expression values. To better infer CCC, we constructed an a priori model. Through a feedforward mechanism, CellGAT constructs a cell communication network and learns the optimal data representation of the model to predict the probability of CCC interactions. CellGAT assigns numerical values to relationships between cell populations, ligands, and receptors, and ranks CCC activities using these embedded communication probabilities. This deep learning framework allows for capturing rich information about cellular signaling networks and transcriptomic data while prioritizing multiple interactions simultaneously. To validate the utility of CellGAT, we apply the model to single-cell transcriptomic data from Drosophila, human heart and lung cancer cell lines, and mouse brain. It is demonstrated that our model can be used to identify downstream activated pathways and biologically relevant changes in CCC in cancer cells following drug interventions and predict cell–cell interactions in spatial microenvironments.

## 2. Materials and Methods

### 2.1. Overview of CellGAT

**Figure 1:**
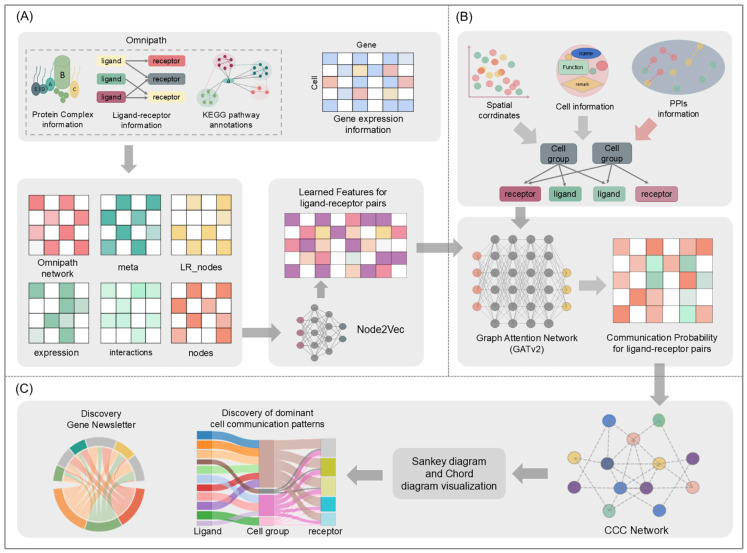
This figure outlines the entire workflow, including data input, model architecture, and analysis steps for predicting intercellular communication. (**A**) Feature construction of CellGAT model input. (**B**) CellGAT predicts interactions. (**C**) CellGAT analyzes prediction results and performs visual analysis.

**Figure 2:**
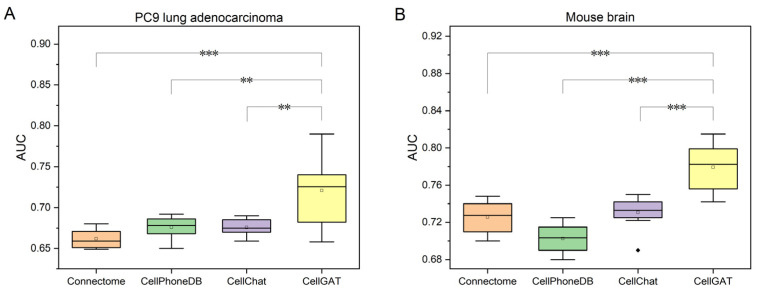
Boxplots comparing the performance of Connectome, CellPhoneDB, CellChat, and CellGAT in predicting intercellular communication. (**A**) Performance on PC9 lung adenocarcinoma dataset. (**B**) Performance on mouse brain dataset. CellGAT achieves significantly higher AUC values compared to other models. (** $\left(p\leq 0.01\right)$, *** (p≤0.001), the discrete points in the figure are one of the results of the test).

Figure 1 provides an overview of the development and testing of CellGAT. CellGAT integrates existing databases’ ligand–receptor information, complex protein information, relevant pathway information, and gene expression information from single-cell transcriptomic data to infer biologically significant known and unknown ligand–receptor interactions between different cell types or cell clusters and visualizes these interactions for analysis. The prediction of cell–cell interactions by CellGAT involves two main steps: (1) constructing a prior model to extract features based on existing ligand–receptor-related information in databases and single-cell sequencing gene expression information (Figure 1A); (2) using transcriptomic information and features output from (1) to predict cell–cell communication in the single-cell dataset (Figure 1B). Finally, based on the computed communication probabilities from (2), CellGAT analyzes the top ten ranked ligand–receptor interactions in terms of communication probability in each single-cell sequencing dataset using Circos and Sankey plots (Figure 1C). CellGAT utilizes gene expression information from single-cell sequencing or spatial transcriptomics data as an input. Furthermore, the output of CellGAT is not a fixed ranked list of interactions predefined by Omnipath between specific cell types. Instead, it is based on the likelihood of ligand–receptor interactions predicted by the model and their corresponding rankings. In this manner, we generate a prediction matrix that reflects potential intercellular interactions. Subsequently, we use the Omnipath database as a validation set to assess the accuracy of the CellGAT predictions.

It is crucial to infer cell–cell communication based on biologically validated ligand–receptor pair-related information. Therefore, in step (1), CellGAT utilizes the OmniPath database, which contains over 30,000 experimentally validated intracellular interactions, more than 3000 intercellular interactions, as well as comprehensive protein information and related pathway information. These pieces of information have been successfully applied in previous methods such as LIANA [13] and CellChat to predict known and unknown cell–cell interactions. CellGAT extracts all significantly expressed ligands and receptors from the input single-cell sequencing gene expression matrix and constructs a directed graph using ligand–receptor pairs existing in the OmniPath database. Additionally, CellGAT provides protein complex and KEGG pathway annotation information in the form of node embeddings. For any pair of ligand and receptor nodes in the directed graph, CellGAT assigns corresponding values to represent the relevance and complexity similarity of ligands/receptors with pathways. Based on these steps, the information in the directed graph is updated, and then feature learning is performed using the Node2Vec [14] framework. This framework updates the representation information of each node based on experimentally validated linkage information.

Gene co-expression is crucial for inferring cell–cell communication. Therefore, in step (2), when computing the communication probability for all possible ligand–receptor pairs, CellGAT employs the Leiden [15] clustering method to identify cell types within a given single-cell sequencing dataset, thereby obtaining cell clusters. For each cluster, CellGAT identifies differentially expressed ligands and receptors. Based on these cell clusters, ligands, and receptors, a new directed graph is constructed. If a ligand/receptor is significantly upregulated in a cell cluster, an edge is drawn from the given cell cluster to the ligand/receptor. Additionally, the previously obtained positional embeddings and new contextual information are further annotated. The newly identified directed graph is then input into the graph attention network (GATv2) [16] for training. GATv2 continuously updates the information of all nodes in a direction favorable to the ligand–receptor interactions based on the learned facts. Finally, the values obtained by GATv2 through inner product calculation serve as the potential communication probabilities for the corresponding ligand–receptor pairs in the single-cell sequencing dataset.

Due to the lack of a universally accepted gold standard for evaluating the accuracy of predicted cell–cell communication, we used the biologically validated OmniPath dataset as a benchmark for assessment. OmniPath, a plugin designed to help researchers analyze and integrate complex molecular and cellular interaction networks, has been extensively studied by Ceccarelli et al., following its creation by Türei et al. These studies have demonstrated the effectiveness of OmniPath in validating communication network analysis. This allowed us to leverage experimentally confirmed LR interactions as a reference point. In addition, we publicly integrated ligand–receptor interaction pairs based on previous scientific studies to validate the performance of CellGAT.

### 2.2. Datasets

CellGAT underwent benchmark testing on four different datasets covering three distinct biological environments: developmental, perturbation, and fibrosis settings. The Drosophila developmental dataset consists of 5835 cells, clustered using the Leiden algorithm (GEO accession number: GSE95025). The PC9 lung cancer cell line perturbation dataset comprises 6778 cells on day 0, 4770 cells on day 7 (replicate 1), and 3515 cells on day 7 (replicate 2), also clustered using the Leiden algorithm (GEO accession number: GSE150949). The mouse brain dataset consists of 2305 cells, clustered using the Leiden algorithm https://data.caltech.edu/records/s0vdx-0k302 (accessed on 25 February 2025). The 10X Visium dataset includes spatial slides of the left ventricle of human myocardial infarction patients, comprising 3100 cells with 9 cell types https://zenodo.org/record/6578047 (accessed on 25 February 2025). To ensure the uniqueness of interaction pairs, we remove duplicates for each ligand–receptor pair within the dataset.

### 2.3. Ligand–Receptor Basic Truth Database

Correctly assigning the roles of signaling molecules and understanding their interactions are crucial for predicting biologically meaningful cell-to-cell communication. Therefore, CellGAT utilizes the OmniPath (1.0.6) [17] database curated by Martina Kutmon et al., which provides all scientifically validated protein–protein interactions (31729 pairs), intercellular interactions (3599 pairs), protein complexes (8022 complexes), and KEGG pathway annotations (7534 entries) [17,18,19] as the experimentally verified relationships to measure the accuracy of the method. These data are presented in tabular form in the OmniPath package in Python (3.11), with ligands and receptors identified using HGCN symbols. We divided the dataset into a training set and a validation set, with the validation set comprising 20% of the original dataset. This ensures that the validation set is a representative subset of the entire dataset, allowing us to assess the performance of our model on unseen data.

### 2.4. Cell Clustering

CellGAT provides a built-in functionality for classifying cells into different cell clusters. Specifically, CellGAT first computes the adjacency relationships between cells, a process that utilizes dimensionality reduction techniques (such as principal component analysis or nearest neighbor embedding) to map high-dimensional gene expression data into a lower-dimensional space. The dimensionality reduction technique used in CellGAT is Node2vec [20], an embedding method specifically designed for graph data. Node2vec not only preserves the neighborhood information of nodes but also retains the structural features of the network, providing an effective tool for calculating the adjacency relationships between cells. Next, the low-dimensional data are subjected to the Leiden community detection algorithm to cluster cells into clusters. The Leiden clustering algorithm automatically determines the number of categories based on the features of the data and assigns cells to the respective categories.

### 2.5. Mapping PPIs to LR Interactions

Given gene expression data $G=\{g_{1},g_{2},...,g_{u}\}$, where $g_{i}$ is the identifier of the *i*-th gene and *u* is the total number of genes, and protein–protein interaction data *P* = {$p_{1},p_{2},...,p_{v}$}, where $p_{j}$ is the identifier of the *j*-th protein and *v* is the total number of proteins, the mapping relationship between ligand or receptor genes and proteins can be represented as a u × v binary matrix *D*, where $D_{ij}$ indicates whether gene $g_{i}$ is associated with protein $p_{j}$. Principle of prioritizing gene co-expression between cells:

(1) $$D_{ij}=\left\{\begin{array}{cc} 1 & \;\mathrm{if}\;g_{i}\;\mathrm{and}\;p_{j}\;\mathrm{are}\;\mathrm{related}\;\\ 0 & \;\mathrm{otherwise}\; \end{array}\right.,$$

The goal of mapping genes to proteins is to determine the elements $D_{ij}$ in the matrix *D*. This can be achieved by comparing the gene names or identifiers from the gene expression data with the protein names or identifiers from the protein–protein interaction data. Specifically, for each gene $g_{i}$ in the gene expression data, it can be checked whether it is associated with any protein $p_{j}$ in the PPI data. If it is, then $D_{ij}$ is set to 1; otherwise, $D_{ij}$ is set to 0.

### 2.6. Data Preprocessing

CellGAT first transposes the gene expression matrix of the input scRNA-seq data and creates an AnnData object. After filtering and normalization of the gene expression information, it calculates the pairwise distances between cells to construct an adjacency matrix. In the process of cell communication, the interactions between cells often rely on the coordinated expression of genes. To adhere to the principle of prioritizing gene co-expression between cells, CellGAT employs the Leiden algorithm for cell clustering on the cleaned data:

(2) $$\begin{array}{c} \vartheta =\frac{1}{2J}\;\sum_{c}\left(e_{c}-\gamma \frac{K_{c}^{2}}{2J}\right), \end{array}$$

Among them, *J* represents the total number of borders, $e_{c}$ represents the number of edges in community node *c*, and $K_{c}$ represents the degree of node *c*. In experiments involving single-cell sequencing data, the cell type annotations are often not provided directly. The Leiden algorithm is widely used in biological network analysis.

For each clustered cell cluster ϑ, the average expression level of genes in each cluster is computed. Finally, information regarding cell types, PPIs, ligand–receptor interactions, and protein complexes is extracted from the OmniPath database. Based on this information, data frames for nodes and interactions are constructed. Subsequently, the data are further organized and processed to generate a dataset containing cells and interactions, which serves as the input for Node2vec node embedding.

### 2.7. Interaction Inference

CellGAT employs two key steps to predict intercellular communication: firstly, it constructs a prior model using the OmniPath database, which encompasses information such as cell types, PPIs, LR interactions, and protein complexes; secondly, it utilizes a graph attention network to infer intercellular communication. The integration of these two steps enables CellGAT to demonstrate outstanding performance in predicting intercellular interactions and offers a comprehensive understanding of the complex communication network among cells. A single ligand–receptor pair refers to the interaction between a specific ligand (a signaling molecule) and its corresponding receptor in the context of cell-to-cell communication. In contrast, true cell-to-cell communication occurs in a complex multicellular environment, where cells interact through multiple signaling pathways and mechanisms. Our study focuses on cell-to-cell interactions within complex multicellular environments, aiming to comprehensively analyze the communication networks between cells, consistent with the definitions of intercellular interactions in the Omnipath database. The interactions considered in this research are directed; for example, we specifically analyze scenarios where one cell type (e.g., cell type A) binds to another cell type (e.g., cell type B) through a receptor–ligand combination (P/L).

### 2.8. Construction of Prior Model

After identifying the ligands and receptors present in the single-cell RNA dataset, cross-referencing is performed using the OmniPath database to determine the valid ligand–receptor interactions present in the dataset. But it is not limited to the connections between each cell group in the cluster. Using the PyTorch [21] Geometric library, a directed graph is constructed with ligands and receptors as nodes, and validation edges are built based on the relationships between ligands and receptors. This directed graph is accompanied by a matrix with dimensions (number of nodes) × (number of nodes). For each cell in the matrix, it represents the connectivity between any two nodes, with its value being

(3) $$\begin{array}{c} W\left(m,n\right)=z_{m,n}+a_{m,n}, \end{array}$$

where *W* represents a cell, and *m* and *n* represent a ligand and its corresponding receptor, respectively. $W\left(m,n\right)$ denotes the value of that cell in the feature matrix, $Z_{m,n}$ is the number of protein complexes where both nodes *m* and *n* are subunits, and $a_{m,n}$ is the number of KEGG pathway members where both nodes *m* and *n* are included.

Next, CellGAT employs the Node2Vec model to perform random walks, training the labeled feature matrix for 100 iterations to generate two-dimensional embeddings. Upon training completion, it extracts the two-dimensional spatial coordinates for each node. Subsequently, it utilizes the inner product of these node positions to construct a position matrix of size equal to the number of ligands multiplied by the number of receptors, thereby forming a spatial distribution map of cell–cell interactions. The closer the distance, the more likely communication exists. Spatial information helps validate that our model favors spatial proximity.

### 2.9. Inference of Intercellular Communication by Graph Attention Network

Before using CellGAT for the prediction of single-cell RNA sequencing data, data undergo logarithmic transformation and normalization. Next, genes with non-negative average expression levels across all cells within a given cell type are identified to determine significantly expressed genes. For each cell group, a directed graph is constructed using Pytorch Geometric, where each cell group points to its respective ligands and receptors. This series of steps lays the groundwork for subsequent predictions of intercellular communication.

The directed graph of the cell group is similar to the graph used when constructing the prior model. Before inference, a feature matrix of size (number of nodes) × (number of nodes) is used to label the directed graph. For nodes *m* and *n*, the value in each cell represents the connection status between any two nodes, as follows:

If node *m* is a ligand/receptor, or node *n* is a ligand/receptor:

(4) $$\begin{array}{c} X\left(m,n\right)=Y_{m}Y_{n}+A_{m,n}, \end{array}$$

where $Y_{m}$ represents the average expression level of ligand/receptor *m*, $Y_{n}$ represents the average expression level of ligand/receptor *n* with respect to $A_{m}$, and *n* corresponds to the value of the matrix generated by the previous step.

If node *m* is a cell group and node *n* is a ligand/receptor:

(5) $$\begin{array}{c} X\left(m,n\right)=Y_{m,n}, \end{array}$$

In the formula, $Y_{m,n}$ is the average expression of ligand/receptor *n* in cell group *m*.

If nodes *m* and *n* both represent cell types, embeddings are provided only when spatial information indicating cell-specific information is included in the dataset. Cell spatial coordinates are an example of this. In such cases, the embedding values are as follows:

(6) $$\begin{array}{c} X\left(m,n\right)=Z_{m,n}, \end{array}$$

where $Z_{m,n}$ is a selection function similar to describing the positional information between cell types m and n. For example, for spatial coordinates, $Z_{m,n}$ is computed as the minimum Euclidean distance between cells of type *m* and cells of type *n*.

(7) $$l\left(h_{m},h_{n}\right)=b^{T}LeakyReLU(O\cdot \left[h_{m}|\left|h_{n}\right]\right),$$

In the equation ${b\in R}^{{2d}^{\prime }}$, ${W\in R}^{d^{\prime }\times d}$ are learnable parameters. For each node *m*, after computing scores with all neighbors, normalization is performed using softmax:

(8) $$\alpha_{mn}={softmax}_{n}\left(l\left(h_{m},h_{n}\right)\right)=\frac{\exp\left(l\left(h_{m},h_{n}\right)\right)}{\sum_{n^{\prime }\in \eta_{m}}\exp\left(l\left(h_{m},h_{n^{\prime }}\right)\right)},\;$$

Finally, GATv2 uses weights for aggregation:

(9) $$h_{m}^{\prime }=\sigma \left(\sum_{n\in \eta_{m}}\alpha_{mn}\cdot Oh_{n}\right),\;$$

For semi-supervised classification tasks such as GATv2, the commonly used loss function is Cross-Entropy Loss, which is formulated as follows:

(10) $$L_{CE}=-\sum_{i=1}^{N}f_{i}\log\left(q_{i}\right),$$

Here, $f_{i}$ represents the true label probability distribution of the *i*-th sample, and $q_{i}$ is the predicted probability that the model assigns to the *i*-th sample being a positive class. This loss function is suitable for situations where nodes have categorical labels, aiming to minimize the difference between the true labels and the predicted labels.

During the training process, the negative log-likelihood loss function is utilized. Upon completion of training, a single forward pass is conducted to obtain two-dimensional embeddings for all nodes in the graph. To identify the top-ranked validated ligand–receptor links, an inner product operation is performed on the embeddings of ligands and receptors, resulting in a matrix of shape (ligands) × (receptors). Subsequently, this matrix is flattened, and the ligand–receptor links are ranked in descending order based on the inner product. To determine the source and destination cell types, CellGAT computes the inner product of cell type embeddings with ligand and receptor embeddings, respectively. This yields a matrix of shape (number of ligands × number of receptors), assigning communication probabilities to each potential edge. The interactions are then sorted based on the inner product values. To avoid randomness of results and verify the stability of the model, we conducted experiments on the data under different default parameters and compared the outcomes.

To delve deeper into the predicted interactions by CellGAT in single-cell transcriptomic data, circular plots and Sankey diagrams were employed to illustrate the top ten results. These graphical tools offer an intuitive display of cellular communication patterns, aiding in unraveling the complexity and significance of intercellular interactions. We visualized the top 10 results, and no impact on accuracy was observed when considering only the top-1, top-3, or top-5 results. The Circos plot focuses on illustrating the overall topology of the cell communication network, highlighting global relationships between different cell types, while the Sankey diagram emphasizes the direction and weight of signal transmission, making it suitable for analyzing dynamic changes in specific pathways. The Circos plot helps identify strong connections among key cell populations, whereas the Sankey diagram traces the detailed flow of signals. The two visualizations complement each other, providing both a global perspective and an in-depth analysis of specific interactions.

All abbreviations used in the text are listed in Abbreviations section.

## 3. Results

### 3.1. Comparison with Other Methods

To better predict intercellular communication, we integrated publicly available LR interaction databases to validate the accuracy of CellGAT. These databases include human and mouse LR interactions based on reliable and validated scientific studies. We prioritized interactions supported in the literature to enhance the accuracy and reliability of predicting intercellular communication. Additionally, we accounted for the presence of multi-subunit complexes, which are critical functional units in complex LR interactions within cellular processes. By considering these factors, we ensured that the predicted interactions reflect the complexity of actual biological processes. Finally, all data were consolidated, and redundant LR pairs were removed to ensure the uniqueness and accuracy of the dataset. As a result, CellGAT retained 5786 validated human LR interactions and 4806 validated mouse LR interactions (Supplementary Data S1 and S2).

To generate negative LR samples, we randomly shuffled ligand and receptor gene lists to create new pairings, ensuring that these randomly generated LR pairs did not overlap with known true interactions. Redundant pairs were eliminated, resulting in a negative sample set for comparative experiments to further validate the model’s performance [22]. Using this approach, we generated negative LR sample sets for the PC9 lung adenocarcinoma and Mouse brain datasets and compared CellGAT with other intercellular communication methods.

The PC9 lung adenocarcinoma dataset is a comprehensive resource for studying high-throughput spatial gene expression in lung adenocarcinoma. Analyzing intercellular communication within this dataset provides insights into cancer cells’ spatial distribution in the lungs or other metastatic target organs, migration pathways, and interactions with other cell types in the microenvironment. This is crucial for uncovering the complex dynamics of the tumor microenvironment, cancer cell metastasis mechanisms, and potential therapeutic targets. We compared the performance of CellGAT with three other methods on this dataset, using their default thresholds and evaluating performance through ACC. As shown in Table 1, CellGAT achieved the highest ACC value.

Next, we evaluated CellGAT on the Mouse brain dataset, a classic resource for studying intercellular communication and signal transduction in the nervous system. Similar to the PC9 dataset, we assessed performance using ACC, and as shown in Table 1, CellGAT again achieved the highest ACC value. These results demonstrate that CellGAT efficiently identifies relevant LR pairs across diverse datasets, consistently exhibiting high accuracy. Furthermore, as shown in Figure 2, CellGAT demonstrates superior performance in predicting intercellular communication, as evidenced by its high AUC values and statistically significant improvements over other models. Its robust framework for integrating validated interaction data is likely the key to its success across diverse biological contexts.

### 3.2. Predicting Pathway Activity in Virtual In Situ Hybridization

**Figure 3:**
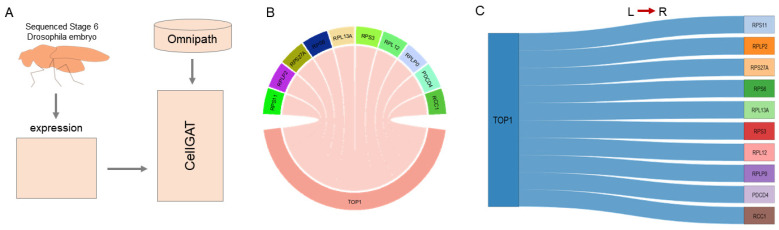
CellGAT applied to sequenced stage 6 Drosophila embryos. (**A**) Sequenced stage 6 Drosophila embryo application diagram. (**B**) Circle diagram visualization of the top ten interaction pairs predicted by CellGAT for sequenced stage 6 Drosophila embryos. (**C**) Sankey diagram visualization of the top ten interaction pairs predicted by CellGAT for sequenced stage 6 Drosophila embryos.

To validate CellGAT’s ability to capture spatial communication patterns in situ hybridization, we applied it to a single-cell sequencing dataset of stage 6 Drosophila embryos, focusing on the pathway activity of CCC (Figure 3A) [23]. Previous studies have identified the expression patterns of the Hippo signaling pathway in stage 6 Drosophila embryos by querying spatially expressed ligands and receptors and reconstructing their spatial expression patterns. These studies further revealed the correlation between Hippo pathway activity and the process of mitosis [24].

CellGAT analyzed the top-ranked CCC interactions in stage 6 Drosophila embryos and visualized them (Figure 3B,C), with the main contribution coming from TOP1. In previous research by Professor Qianwen Sun and colleagues, TOP1 activity was found to influence R-loop levels, and the topological organization of the genome is highly correlated with activities such as DNA replication, transcription, and repair. CellGAT not only identified CCC interactions with members of the Hippo pathway as ligands/receptors but also discovered interactions with many other pathways, including animal mitosis, MAPK signaling, the cell cycle [25], phospholipase D signaling, and the Notch signaling pathway. MAPK, phospholipase D, and Notch signaling have been confirmed to participate in Drosophila embryonic development. While CellGAT’s results cannot determine the downstream effects of ligand–receptor interactions, the identified interaction between the cell cycle and the Hippo signaling pathway may be relevant to previous research findings.

Finally, CellGAT compared the coverage of the Hippo pathway in the top 1000 interactions with other CCC methods. Among the three CCC methods—CellPhoneDB [9], Connectome [26], and CellChat [12]—none captured any interactions involving the Hippo pathway. The reason for this outcome might be that other CCC methods only utilize intercellular interactions, whereas CellGAT was trained on all intra- and intercellular interactions. Although previous studies have identified interactions associated with the ErbB pathway and found its involvement in the Hippo pathway in Drosophila [27], experimental evidence suggests that CellGAT uniquely predicts previously published cell–cell communication results and provides more intricate interactions.

### 3.3. Predicting Changes in Intercellular Communication Caused by Non-Genetic and Pharmacological Perturbations

**Figure 4:**
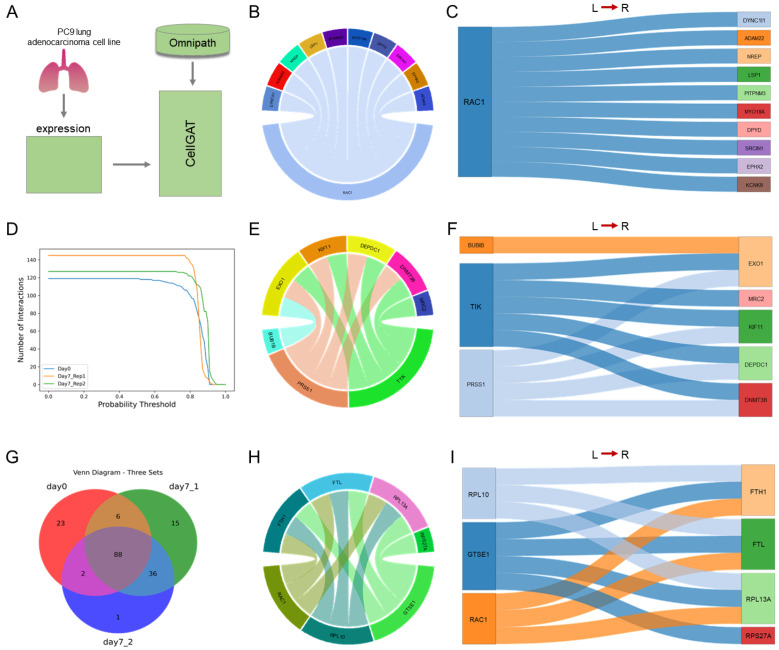
CellGAT applied to PC9 lung adenocarcinoma cell line dataset. (**A**) PC9 lung adenocarcinoma cell line dataset application diagram. (**B**,**C**) Visualization of the circle diagram and Sankey diagram of the top ten interaction pairs predicted by CellGAT for day 0 dataset. (**D**) Changes in the interaction logarithm of day 0, day 7, and day 14 datasets with different probability thresholds. (**E**,**F**) Visualization of the circle diagram and Sankey diagram of the top ten interaction pairs predicted by CellGAT for day 7 dataset. (**G**) Venn diagram visualizes the overlap of predicted interaction pairs by CellGAT in three datasets of day 0, day 7, and day 14 dataset. (**H**,**I**) Visualization of the circle diagram and Sankey diagram of the top ten interaction pairs predicted by CellGAT for day 14 dataset.

We applied CellGAT to study CCC in a dataset covering PC9 lung adenocarcinoma cells before and after drug treatment (Figure 4A), aiming to explore CellGAT’s potential in revealing the effects of drug treatment on pathways and CCC [28]. CellGAT inferred cell–cell communication in populations of cells treated with osimertinib (a tyrosine kinase inhibitor) [29] at days 0, 7, and 14, and visualized them, respectively, for day 0 (Figure 4B,C), day 7 (Figure 4E,F), and day 14 (Figure 4H,I). To validate CellGAT’s ability to further identify drug targets/mechanisms of action [30], we categorized these data into two classes: (1) the same biological sample under different conditions (day 0, 7/14); (2) the same biological replicate sample (day 7, 14). As shown in Figure 4, CellGAT yielded results on three independent datasets under two different conditions, including one single-cell RNA dataset for day 0 and two biological replicate single-cell RNA datasets for day 7.

Based on the LR interactions validated in the OmniPath database and known pathway information annotated through KEGG, CellGAT predicted the number of ligand–receptor pairs in the two classes of datasets, as shown in Figure 4D. In the dataset for day 7, the second-ranked ligand was found to be TNF, a multifunctional pro-inflammatory cytokine belonging to the tumor necrosis factor (TNF) superfamily. In previous scientific research, elevated levels of TNF expression have been observed in lung cancer and may play a tumorigenic role [31,32]. We analyzed the top 100 validated CCCs in each of the three datasets, and the overlap of the predicted results for the three datasets is shown in in Figure 4G. In both classes of datasets, CellGAT discovered not only different interactions in the same biological sample before and after treatment but also differences in the predicted results for the same biological replicate sample after treatment. This discrepancy demonstrates CellGAT’s ability to predict new interactions in the same dataset, which may suggest new findings regarding drug sensitivity or chemotherapy resistance in PC9 [33].

**Figure 5:**
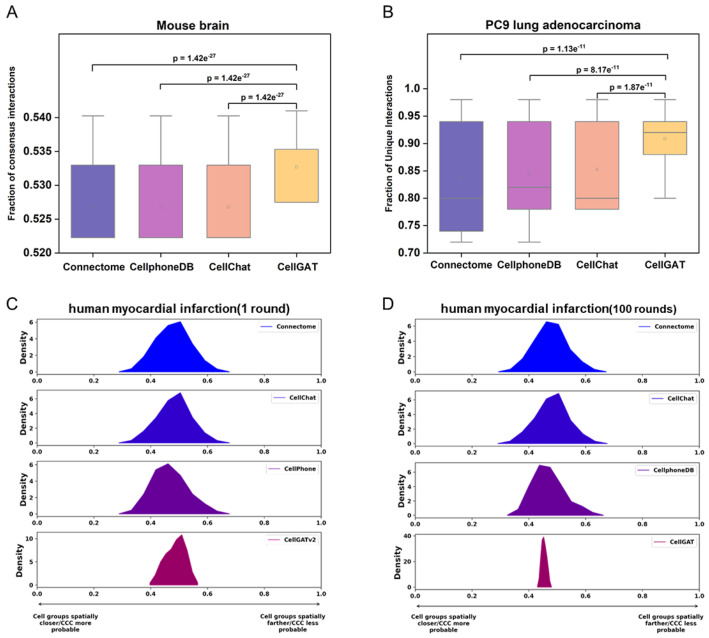
CellGAT compared with other methods. (**A**) Comparison of the performance of CellGAT with Connectome, CellphoneDB, and CellChat in predicting verified cell–cell interaction pairs in the Mouse brain dataset. (The means are 0.527, 0.527, 0.527, and 0.533, respectively.) (**B**) Comparison of the performance of CellGAT with Connectome, CellphoneDB, and CellChat in predicting validated interaction pairs in the PC9 lung adenocarcinoma dataset. (The means are 0.80, 0.82, 0.81, and 0.91, respectively.) (**C**,**D**) Comparison of the spatial proximity of CellGAT in predicting validated interaction pairs with Connectome, CellphoneDB, and CellChat in the human myocardial infarction dataset.

To evaluate the ability of the method to explore novel interactions, we compared the proportion of non-overlapping results across the three datasets. In comparison with other methods, CellGAT captured a relatively higher proportion of unique interactions among the top 100 biologically validated interactions, as shown in Figure 5B. This indicates that CellGAT is better able to identify unique interactions before and after disease treatment. Therefore, CellGAT can identify unique interactions before and after tumor treatment, potentially providing new insights for drug therapy.

### 3.4. Predicting Neuronal Cell Interactions

**Figure 6:**
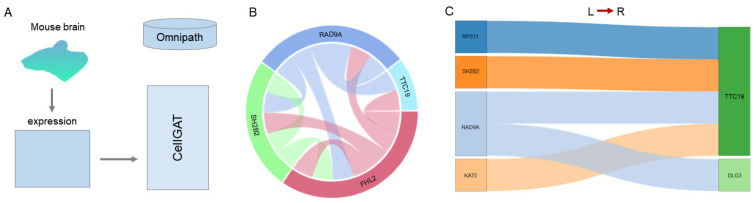
CellGAT applied to Mouse brain dataset. (**A**) Mouse brain dataset application diagram. (**B**) Circle diagram visualization of the top ten interaction pairs predicted by CellGAT for Mouse brain dataset. (**C**) Sankey diagram visualization of the top ten interaction pairs predicted by CellGAT for Mouse brain dataset.

Communication between neurons plays a critical role in cognitive processes and behavioral performance. By delving into the communication networks among neurons, we can uncover patterns of connectivity between different brain regions and understand how these connections influence cognitive function and behavior. To validate the ability of CellGAT to capture communication patterns between neurons, we applied it to a mouse brain dataset (Figure 6A). We curated a validation set, called consensus_mouse, based on interaction pairs and pathway information from the OmniPath database in this dataset.

Therefore, CellGAT further analyzed the top-ranked interaction pairs and visualized them (Figure 6B,C). We discovered an interaction between RPS11 and TTC19, where previous research has demonstrated RPS11 as an antibody, while mutations in TTC19 lead to neural damage [34]. Additionally, in Burkitt’s lymphoma cell lines, SH2B2 is phosphorylated on tyrosine upon B-cell receptor stimulation. SH2 domains typically bind ligands relative to phosphotyrosine. Its association with stimulation and Grb2 is independent, and it appears to play a role in signaling pathways from the receptor to Shc/Grb2 upon receptor activation. These interactions are likely associated with the pathogenesis of certain diseases. Previous studies have indicated that the interaction between SH2B2 and TTC19 involves the coupling of immune receptors with the Ras signaling pathway. Upon activation of immune receptors during immune response, a cascade of signaling events, including activation of the Ras signaling pathway, is initiated. SH2B2, as a crucial signaling adaptor protein, likely mediates the signal transduction of immune receptors by binding to phosphorylated tyrosine residues on active receptors and interacts with TTC19 during this process. TTC19, as a protein, may regulate the formation or function of this complex, further impacting the progression of signal transduction. This interaction likely plays a pivotal role in modulating cellular immune responses, especially in regulating the Ras signaling pathway. Therefore, the findings from CellGAT provide a novel perspective on inter-neuronal communication, further enhancing our understanding of the regulatory mechanisms underlying neuronal function.

Based on a meticulously planned validation set, we compared the performance of CellGAT with other methods on the Mouse brain dataset (Figure 5A). The results demonstrate that CellGAT outperforms current methods used to predict interactions between neurons and exhibits greater stability. This suggests that CellGAT effectively identifies interactions between neurons, potentially providing opportunities for discovering new drug targets. “Verified interaction pairs” refer to the biologically validated dataset from OmniPath. OmniPath is a resource designed to assist researchers in analyzing and integrating complex molecular and cellular interaction networks. Following its creation by Türei et al., it has been extensively studied by Ceccarelli et al., demonstrating its effectiveness in validating communication network analyses. Additionally, in our methodology, “interaction pairs” specifically denote the intercellular interaction pairs predicted by CellGAT, which align with the known biological interaction information from the OmniPath database. By comparing our model’s predictions against existing, experimentally validated interaction pairs in OmniPath, we validate the accuracy and reliability of our predictions.

### 3.5. Predict Spatially Adjacent CCC

**Figure 7:**
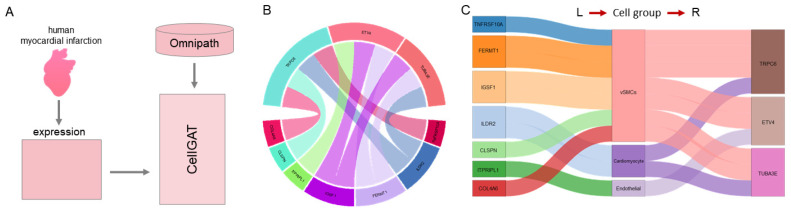
CellGAT applied to human myocardial infarction dataset. (**A**) Human myocardial infarction dataset application diagram. (**B**) Circle diagram visualization of the top ten interaction pairs predicted by CellGAT for human myocardial infarction dataset. (**C**) Sankey diagram visualization of the top ten interaction pairs predicted by CellGAT for human myocardial infarction dataset.

To assess CellGAT’s ability to predict interactions consistently with other modalities and to analyze new directions in CCC, CellGAT was applied to a dataset of human myocardial infarction, which includes spatial transcriptomic data from different regions affected by myocardial infarction (such as the ischemic zone, remote zone, border zone, and fibrotic zone), as well as unaffected control regions (Figure 7A) [35]. The human myocardial infarction 10X Visium spatial transcriptomics dataset we used includes samples from the fibrotic zone (FZ). The primary objective of this benchmark test is to demonstrate CellGAT’s ability to learn graph structures consistent with spatial microenvironments, namely cellular localization and proximity. This provides a new perspective for comparing its prediction results with background knowledge of intercellular communication. For the sequencing data from various regions of the heart, cellular expression data are accompanied by spatial coordinates. Besides validating expected ligand–receptor pairs, CellGAT identifies potential spatial proximity between ligand cell populations and receptor cell populations, influencing the likelihood of these interactions.

We applied a single spatial transcriptomic data sample from the fibrotic zone (FZ) to infer intercellular communication using CellGAT (Figure 7B,C). The dataset contains spatial coordinates for all cells, used to construct maps organized by cell types. We utilized this map to partition subsets of cell types involved in interactions and visualize their spatial proximity (Figure 5C,D). In the prediction results, we identified a ranked-sixth interaction involving vascular smooth muscle cells (vSMCs) and myeloid cells [36]. This interaction has been studied in the carotid artery region, involving interactions between CXCL12 and CXCR4. Previous studies have extensively analyzed this interaction, particularly to understand its role in myocardial infarction, demonstrating its high biological relevance to the dataset [37]. Additionally, we identified lower-ranked cell–cell interactions involving peripheral cells and neurons. Within these cell populations, we observed a ligand–receptor interaction between RELN and ITGB1. This validated interaction primarily occurred in glioblastoma cells. RELN is a critical signaling protein known for its role in brain neurodevelopment and neuronal migration. ITGB1 is an integrin protein that serves as a key link between the extracellular matrix and cells. It is speculated that RELN may mediate cell–cell signaling, cell migration, or other related biological processes in glioblastoma cells by binding to its receptor ITGB1.

When comparing the spatial proximity of the top 10 predicted interactions by CellGAT with other methods in the human myocardial infarction dataset, CellGAT exhibits lower mean and variance values. These findings suggest that, based on accurate information, CellGAT not only prioritizes biologically meaningful and validated interactions but also prioritizes interactions between cell types that are spatially adjacent.

## 4. Discussion

In this study, we introduce a novel bioinformatics method called CellGAT, designed to infer intercellular communication from single-cell RNA sequencing data and validate it using transcriptomic data. This method integrates single-cell gene expression data with ligand–receptor pair databases and previous biological pathway annotations to achieve a more comprehensive and accurate inference of intercellular communication. The scope of the model extends beyond connections between cell groups within individual clusters to encompass broader interactions across the entire cellular network. This interconnectedness is crucial for understanding the complex dynamics of cell communication, as it enables signal propagation and coordination of cellular activities throughout the tissue or organism. By considering these broader interactions, the model aims to provide a comprehensive perspective on cell communication patterns, uncovering new insights and patterns that may not be evident within individual clusters.

CellGAT utilizes a directed graph representation based on graph neural networks to depict the relationships between cell types and identified ligand–receptor pairs. Additionally, it prioritizes information on cell–cell interactions and interprets aspects such as cell localization, pathway annotations, and protein complexes. During the training phase, CellGAT utilizes PPIs obtained from the OmniPath database to explain CCC occurring between cells/cell types and internally. By training on both intra- and inter-cellular PPIs obtained from the OmniPath database, CellGAT can predict CCC between cell types and internally. To validate CellGAT’s ability to predict intercellular communication, we utilized four datasets and compared them with three other methods. In the fly dataset, CellGAT’s inferred intercellular interactions were compared with validated results, demonstrating its predictive capability for intercellular communication. For the other three datasets, we focused on intercellular communication to reflect the definition used in previous studies and emphasized more diverse patterns occurring between different cell types/populations. We were able to demonstrate that CellGAT can reproduce intercellular communication results seen through spatial reconstruction, identify unique interactions in cell populations after drug treatment, and prioritize interactions occurring between spatially adjacent cell types.

Our model effectively integrates prior knowledge from databases such as OmniPath, enabling accurate inference of cell–cell communication based on known ligand–receptor pairs. By combining multi-omics data with advanced computational methods, such as node embedding and graph attention networks, we provide a robust platform capable of predicting interactions across various biological contexts. However, the model’s reliance on existing databases like OmniPath introduces certain limitations, particularly when predicting novel cell–cell interactions. To address this issue, researchers are exploring more data-driven approaches, leveraging large-scale single-cell and spatial transcriptomics datasets to reduce dependency on existing databases, discover novel ligand–receptor interactions, and enhance the model’s comprehensiveness.

Exploring intercellular communication offers new insights into biomedical research, deepening our understanding of interactions within and between cells. By delving into the interactions between different cell types, developmental stages, disease states, or external perturbations, we can uncover crucial information closely related to cell state recognition and functional regulation. Although many methods exist to predict potential communication activities between cells using validated ligand–receptor databases and transcriptomic data, these approaches often struggle to fully capture the complexity of cellular social networks and face limitations in integrating different modalities. Preliminary research findings suggest that for more accurate prediction of intercellular communication, we need to consider the complexity of intercellular interaction networks. This involves a comprehensive understanding of intercellular signal transduction and how to integrate this information with prior knowledge of ligand–receptor interactions. Therefore, studying CCC requires more comprehensive and in-depth methods to better understand the complex interaction networks between cells.

Our model effectively integrates prior knowledge from existing databases such as OmniPath, enabling highly accurate inference of cell–cell communication based on known ligand–receptor pairs. However, differences in cell communication across tissue samples may exist, and certain unique interaction pairs in cancer tissues could limit the model’s accuracy when applied to other datasets. Additionally, the lack of standardized datasets to measure the accuracy of these predictions restricts the direct comparison between methods, as well as the reproducibility and comparability of their performance. The development of multi-omics datasets in the future may help address this limitation. Furthermore, there is currently no concrete definition of cell communication. Communication types include intracellular and extracellular, long-range, and short-range signaling. Integrating a unified definition of cell communication could provide researchers with clearer directions, allowing them to overcome existing limitations and further advance the field.

Furthermore, in the inference of intercellular communication, the exacerbation due to the lack of foundational facts has been previously recognized, emphasizing the critical importance of speculative analysis based on foundational facts. can lead to the discovery of more effective CCC interactions in different contexts, thereby improving the prediction and evaluation of ligand–receptor interactions. Based on these facts, we developed a graph-based deep learning method, CellGAT, capable of accurately capturing ligand–receptor communication in single-cell transcriptomic data. Additionally, when studying spatially regulated intercellular communication, integrating different omics data and multimodal datasets (such as 10× Multiome and Digital Spatial Profiling) can provide more comprehensive, multidimensional information, enhancing our understanding of cell interactions and regulatory mechanisms. In such cases, reliable computational models are needed to accurately integrate multimodal data and perform inference.

## 5. Conclusions

In summary, CellGAT combines gene expression data with protein–protein interactions (PPIs) and protein complex information from the OmniPath database. This approach helps to mitigate the loss of predictive accuracy due to the inactivation of ligands or receptors caused by certain gene inactivation. Consequently, CellGAT demonstrates high robustness and accuracy when handling data with abnormal gene expression. CellGAT integrates node embedding algorithms with graph attention networks, excelling at capturing both local and global graph structural features within the data. The node embedding algorithms effectively extract graph structure information, while the graph attention networks adeptly process these features, making CellGAT highly effective in predicting cell interactions.

You can access the open-source Python implementation of CellGAT on GitHub at https://github.com/wuzhenao/CellGAT (accessed on 25 February 2025). This version provides tutorials and examples for the following analyses: ligand activity analysis based on a gene set of interest, single-cell ligand activity analysis, and visualization of ligand-to-target signaling pathways. Additionally, we offer tutorials for model validation, including training the prior model with databases and evaluating target gene and ligand activity. Users can utilize these tutorials to compare their methods with CellGAT and build models tailored to their specific needs (for example, using their own data sources to explore more cell–cell communications).

## Figures and Tables

**Table 1:** Comparison of CellGAT and other three methods ACC.

	CellGAT	CellPhoneDB	CellChat	Connectome
PC9_lung_adenocarcinoma	**0.91**	0.85	0.80	0.87
Mouse_brain	**0.85**	0.78	0.81	0.79

## Data Availability

Sequencing data for the sixth stage of Drosophila embryo can be found in the GEO database under accession code GsSE95025. The data for PC9 cell line treated with osimertinib before and after treatment are available under the GEO registration code GSE150949. The Mouse brain dataset can be obtained from “https://data.caltech.edu/records/s0vdx-0k302 accessed on 25 February 2025”. Additionally, spatial transcriptomics data can be accessed from “https://zenodo.org/record/6578047 accessed on 25 February 2025”.

## Supplementary Materials

The following supporting information can be downloaded at https://www.mdpi.com/article/10.3390/biom15030342/s1, Supplementary S1: Supplementary_Data_1.csv, Supplementary S2: Supplementary_Data_2.csv.

## Abbreviations

CCC	cell-cell communication
LR	ligand-receptor
PPIs	protein-protein interactions
